# A consideration of the relative contributions of different microbial subpopulations to the soil N cycle

**DOI:** 10.3389/fmicb.2012.00373

**Published:** 2012-10-23

**Authors:** Peter J. Bottomley, Anne E. Taylor, David D. Myrold

**Affiliations:** ^1^Department of Crop and Soil Science, Oregon State UniversityCorvallis, OR, USA; ^2^Department of Microbiology, Oregon State UniversityCorvallis, OR, USA

**Keywords:** ammonium consumption, dominant and rare taxa, growth active subpopulations, N immobilization, N mineralization, nitrification, nitrogen cycling, substrate-responsive subpopulations

## Abstract

We examine and discuss literature targeted at identifying “active” subpopulations of soil microbial communities with regard to the factors that affect the balance between mineralization and immobilization/assimilation of N. Whereas a large fraction (≥50%) of soil microbial biomass can immediately respire exogenous substrates, it remains unclear what percentage of both bacterial and fungal populations are capable of expressing their growth potential. The factors controlling the relative amounts of respiratorily responsive biomass versus growth-active biomass will impact the balance between N mineralization and N immobilization. Stable isotope probing of *de novo* DNA synthesis, and pyrosequence analyses of rRNA:rDNA ratios in soils have identified both numerically dominant and rare microbial taxa showing greatest growth potential. The relative growth responses of numerically dominant or rare members of a soil community could influence the amount of N immobilized into biomass during a “growth” event. Recent studies have used selective antibiotics targeted at protein synthesis to measure the relative contributions of fungi and bacteria to ammonification and ${NH}_{4}^{+}$ consumption, and of NH_3_-oxidizing archaea (AOA) and bacteria (AOB) to NH_3_ oxidation. Evidence was obtained for bacteria to dominate ${NH}_{4}^{+}$ assimilation and for fungi to be involved in both consumption of dissolved organic nitrogen (DON) and its ammonification. Soil conditions, phase of cropping system, ${NH}_{4}^{+}$ availability, and soil pH influence the relative contributions of AOA and AOB to soil nitrification. A recent discovery that AOA can ammonify organic N sources and oxidize it to ${NO}_{2}^{-}$ serves to illustrate roles for AOA in both the production and consumption of ${NH}_{3}/{NH}_{4}^{+}$. Clearly, much remains to be learned about the factors influencing the relative contributions of bacteria, archaea, and fungi to processing organic and inorganic N, and their impact on the balance between mineralization and immobilization of N.

## INTRODUCTION

**FIGURE 1:**
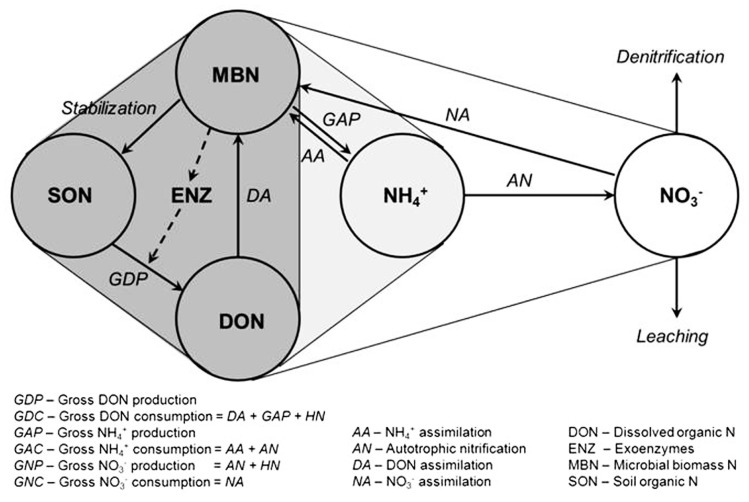
**Diagram of the microbial N cycle in aerobic soil**. Major pools of N are shown in circles, major fluxes by solid arrows, and the dashed arrow represents the production of exoenzymes (e.g., proteases) for the depolymerization of soil organic N. Pools and fluxes in dark gray relate to turnover of dissolved organic N, those in light gray relate to turnover of ${NH}_{4}^{+}$, and unshaded to turnover of ${NO}_{3}^{-}$. Based on Myrold and Bottomley (2008).

The soil N cycle consists of several N pools and inter-connected transformations (**Figure 1**). Microbial biomass is central to the processes involved in producing and immobilizing inorganic N. Furthermore, microbial biomass contributes directly to the pool of soil organic N through its death and turnover. The placement of soil microbial diversity and dynamics into context with biological functions associated with the N cycle still remains a grand challenge (Myrold and Bottomley, 2008; Strickland et al., 2009; McGuire and Treseder, 2010). Conservation of soil N depends on the maintenance of a balance between the rate of depolymerization of organic N, the portion of it that is mineralized to ${NH}_{4}^{+}$, and the rate of ${NH}_{4}^{+}$ consumption by three different sinks: (a) plant growth, (b) heterotrophic microbial assimilation, and (c) NH_3_-oxidizing bacteria (AOB) and archaea (AOA). Simplistically speaking, when sink (c) is larger than (a) plus (b), ${NO}_{3}^{-}$ accumulates and becomes vulnerable to leaching and/or denitrification from the ecosystem. It is the microbiology underpinning these alternate fates of ${NH}_{4}^{+}$ that is the framework of this review.

### HOW DOES GROSS MINERALIZATION OF N (N_min_) AND GROSS IMMOBILIZATION OF N (N_assim_) OCCUR IN THE SAME SOIL VOLUME?

**FIGURE 2:**
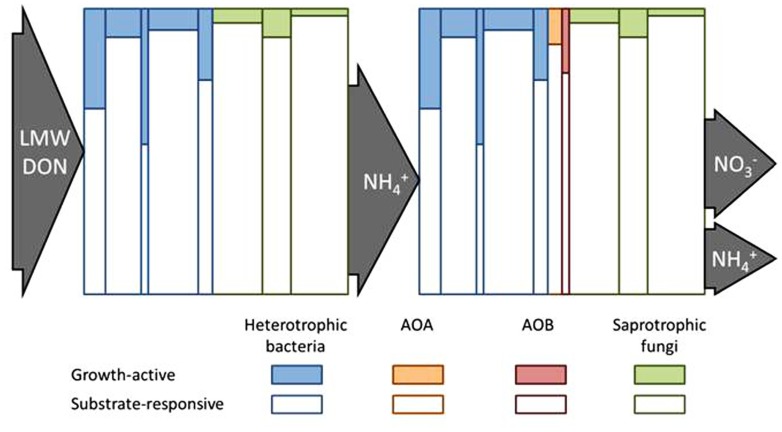
**Conceptual diagram to illustrate how the fate of soil N pools might be controlled by the growth-active fraction (GAF) of the substrate-responsive population (SRP) of microorganisms**. The heights of the individual vertical columns represent the SRP of different taxa and their widths represent the relative sizes of each taxa as part of the whole microbial community. The shaded portion of each column represents the GAF of each SRP taxon. The left side depicts that low molecular weight (LMW) dissolved organic N (DON) is taken up and metabolized by the SRP but N is only assimilated by the GAF. The balance between ${NH}_{4}^{+}$ mineralized versus N immobilized will be influenced by the relative amounts of GAF versus SRP and determine the net ${NH}_{4}^{+}$ mineralized. The right panel depicts that growth-active heterotrophs and NH_3_-oxidizing archaea (AOA) and bacteria (AOB) will compete for ${NH}_{4}^{+}/{NH}_{3}$ with the outcome being affected by the GAF/SRP ratio of the ${NH}_{4}^{+}/{NH}_{3}$ assimilating heterotrophs (assuming that AOA and AOB only assimilate a small fraction of ${NH}_{4}^{+}$ consumed) and by the relative sizes of and kinetic properties of the SRP populations of AOA and AOB. A similar panel could be drawn to show the assimilation of ${NO}_{3}^{-}$ by the GAF of heterotrophic microorganisms.

For >50 year soil scientists have measured fluxes of ${NH}_{4}^{+}$ production and consumption in soils using the ^15^N isotope pool dilution approach (Kirkham and Bartholomew, 1954; Davidson et al., 1991; Hart et al., 1994; Murphy et al., 2003). Gross rates of N mineralization in soil are positively correlated with total soil C and N contents and the size of the microbial biomass pool (Booth et al., 2005). Furthermore, gross rates of microbial ${NH}_{4}^{+}$ and ${NO}_{3}^{-}$ assimilation are positively and linearly related to gross N mineralization rates. The fact that heterotrophic ${NH}_{4}^{+}$ assimilation (N_assim_) can be a sink of substantial magnitude in the same soil volume where ${NH}_{4}^{+}$ is also being produced by mineralization (N_min_) has prompted a variety of explanations over the years. Clearly, the balance between N_min_ and N_assim_ will be influenced by the extent that microbial growth (N_assim_) is coupled to N_min_ (**Figure 1**). For example, it has been proposed that N_min_ and N_assim_ processes are carried out concurrently by physically separated microbial populations growing on different C sources of different C:N ratios (Schimel and Bennett, 2004; Schimel and Hattenschwiler, 2007; Manzoni et al., 2008). Co-existing metabolisms of different microbial groups, such as fungi and bacteria, with different C and N spectra usage and growth efficiencies might also influence the balance between N_min_ and N_assim_ in the same soil volume (Boyle et al., 2008; Rinnan and Baath, 2009). **Figure 2** represents an attempt to illustrate how physiological heterogeneity among soil microbial subpopulations caused by varying degrees of starvation/dormancy might influence their relative demands for C and N (Schimel et al., 2007; Allison et al., 2010; Dworkin and Shah, 2010; Lennon and Jones, 2011), as might antagonistic competition between bacteria and fungi for growth resources (Rousk et al., 2008, 2010b). Finally, the relative portions of dissolved organic nitrogen (DON) ammonified/assimilated will depend upon the relative needs of the microbial populations for C and N. For example, it has been shown that soil proteolytic activity can be increased by N limitation and decreased by increased ${NH}_{4}^{+}$ availability illustrating the well accepted role of soil proteins as N sources (Sims and Wander, 2002; Allison and Vitousek, 2005). Yet, proteolytic activity can also be repressed by addition of glucose (Geisseler and Horwath, 2008) suggesting a role for DON as a C source.

### SUBSTRATE-RESPONSIVE POPULATION: GROWTH READY OR NOT?

Whereas it is well accepted that the vast majority (>80%) of soil microbes probably reside in some state of dormancy, several studies have shown that at least one-half of soil microbial populations are respiratorily active (Jones and Lennon, 2010; Lennon and Jones, 2011; Hobbie and Hobbie, 2012). Furthermore, it is well known that soil respiration responds rapidly (within 1–3 h) to the addition of exogenous C substrates, including amino acids (Jones and Murphy, 2007; Jones et al., 2009). This response is the basis of the well-established substrate-induced respiration (SIR) assay used to measure soil microbial biomass (Anderson and Domsch, 1978). In SIR the rule-of-thumb is that 40 μg of respiratorily active soil biomass C generates a respiratory response of ~0.48 μg CO_2_-C/h. With this relationship in mind, a recent series of papers by Rousk et al. (2008, 2009, 2010b) focused on factors influencing the relative contributions of fungi and bacteria to soil activities. They determined that substrate-induced soil microbial biomass ranged between 100 and 200 μg microbial biomass C/g soil which would support a substrate-induced rate of respiration of ~1.3–2.6 μg CO_2_-C/g soil/h or (~30–60 μg CO_2_-C/g soil/day). The bacterial and fungal growth potentials of the same soils were measured by following ^3^H-leucine incorporation into hot trichloroacetic acid (TCA) precipitable material (presumed to be protein), and ^14^C acetate incorporation into ergosterol, respectively. The rates of leucine assimilation ranged between 10 and 100 pmol/g soil/h. We have attempted to extrapolate from these data to obtain a rate of N assimilation into protein. We used the following correction factors/assumptions, that ~50% of assimilated leucine is incorporated into protein, and that only 10% of the total soil bacterial population is extracted and represented in the soil slurry assay (Baath, 1994, 1998; Rousk, personal communication). We further assumed that leucine incorporation into protein tracks total protein synthesis, that leucine represents ~8–10% of microbial cell protein (Kirchman et al., 1985; Neidhardt et al., 1990), and that the average N content of protein is 16%. These rates of leucine incorporation extrapolate to ~0.28–2.8 μg N assimilated/g soil/day. In the case of fungal growth potential, the rates of acetate incorporation into ergosterol (C_28_H_44_O, mw = 396) ranged between 10 and 80 pmol acetate incorporated/g soil/h, equivalent to ~0.7–5.7 pmol ergosterol synthesized/g soil/h (assuming a minimum of 14 acetate molecules required per molecule of ergosterol), and ~ 6.8–54.4 ng ergosterol synthesized/g soil/day. Using the authors’ biomass conversion factor (5 ng ergosterol/1 μg fungal biomass), the ergosterol synthesis rate extrapolates to 1.3–10.9 μg fungal biomass formed/g soil/day. Assuming that fungal biomass is 45% C and has a C:N of 10:1 this growth rate is equivalent to ~0.06–0.48 μg N immobilized/g soil/day. From the sum of the leucine/ergosterol assays the rate of N assimilation ranges from 0.34 to 3.4 μg N/g/day. These values fall within the range of ${NH}_{4}^{+}$ assimilation rates that have been measured in whole soils by ^15^N isotope pool dilution (0.1–10 μg ${NH}_{4}^{+}$-N/g/day; Booth et al., 2005). Another approach to this issue is to consider that the substrate-responsive biomass of 100–200 μg C/g soil has the potential to respire exogenous C at a rate of 30–60 μg CO_2_-C/g/day. If this rate of respiration is coupled to C assimilation at a yield efficiency of 50%, then 30–60 μg C are assimilated/g/day. If bacteria were solely responsible for the C assimilation with a C:N of 5:1, this extrapolates to 6–12 μg N assimilated/g/day. Alternately, if fungi were solely responsible with a C:N 10:1, the rate equals 3–6 μg N assimilated/g/day. This range of potential N assimilatory values (3–12 μg N/g/day) falls within the range of ${NH}_{4}^{+}$ assimilation rates mentioned above; it is somewhat higher than the range of N assimilation estimates calculated from the leucine incorporation and ergosterol synthesis data.

Important unanswered questions are: (1) to what extent does the dormant, substrate-responsive microbial population actually process DON and contribute to N_min_? (2) How quickly does a substrate-responsive population transition into a N assimilatory sink and shift the N_min_:N_assim_ balance? (3) How well does the leucine plus acetate assimilatory subpopulations of bacteria and fungi detected in the Rousk et al. (2008, 2009, 2010b) studies represent the ${NH}_{4assim}^{+}$ sink routinely detected in ^15^N isotope pool dilution whole soil studies? (4) Or, does the leucine incorporating bacterial population represent a DON assimilating subpopulation that might be different from the ${NH}_{4}^{+}$ assimilating subpopulation?

### WHAT CONTROLS THE OUTCOME OF COMPETITION FOR ${NH}_{3}/{NH}_{4}^{+}$ BETWEEN HETEROTROPHS AND NH_3_ OXIDIZERS, AND AMONG DIFFERENT NH_3_ OXIDIZERS?

It has been shown that the extent of ${NO}_{3}^{-}$ leaching from arable and grassland soils correlates well with the ratio of the rate of N nitrification/rate of N immobilized (N/I; Stockdale et al., 2002). Yet, the factors underpinning the relative rates of N and I and the range of magnitude of N/I ratio are not well understood. For example, gross nitrification rates can consume the majority of ${NH}_{4}^{+}$ mineralized at low N mineralization rates (<2 μg N/g soil/day). However, as the rate of N mineralization increases and both ${NH}_{4}^{+}$ assimilation and nitrification increase, the latter consumes a disproportionately lower percentage of ${NH}_{4}^{+}$ than the former (Booth et al., 2005). A combination of increased C availability and a higher affinity for ${NH}_{3}/{NH}_{4}^{+}$ has been proposed to play a role in the relative success of heterotrophic microorganisms over AOB (Kleiner, 1981; Chen and Stark, 2000). Heterotrophic bacteria have an affinity for ${NH}_{4}^{+}$ that ranges between 3.7 and 13.2 μM (Reay et al., 1999). In contrast, the affinity for ${NH}_{4}^{+}$ by strains of *Nitrosospira*, the dominant genus of soil AOB, ranges from 78 to 590 μM ${NH}_{4}^{+}$ (Jiang and Bakken, 1999; Bollmann et al., 2005; Taylor and Bottomley, 2006) implying that AOB might compete poorly with heterotrophs for ${NH}_{4}^{+}$ under soil conditions with adequate labile C. On the other hand, it has been observed that heterotrophic assimilation of ${NH}_{4}^{+}$ was less than ${NO}_{3}^{-}$ assimilation, suggesting that NH_3_ oxidizers can compete successfully with heterotrophs for ${NH}_{4}^{+}$ (Burger and Jackson, 2003). To add more complexity to this topic, evidence was recently obtained that the thaumarchaeal community of an acidic peat soil (pH range 3.8–4.7) preferentially oxidized ${NH}_{4}^{+}/{NH}_{3}$ generated from organic N sources over exogenous ${NH}_{4}^{+}$ (Levicnik-Hofferle et al., 2012). One interpretation of these results is that AOA are involved in the direct uptake of DON, and that oxidation of NH_3_ occurs after intracellular deamination of DON. This strategy of ${NH}_{3}/{NH}_{4}^{+}$ oxidation permits thaumarchaea to circumvent direct competition with heterotrophs for soil ${NH}_{4}^{+}$ (but not for DON),and provides further evidence of mixotrophic metabolism of thaumarchaeal ${NH}_{3}/{NH}_{4}^{+}$ oxidizers under soil conditions (Tourna et al., 2011). Evidence has been obtained recently for both the marine AOA “*Candidatus* *Nitrosopumilus maritimus*” strain SCM1 and a soil AOA “*Candidatus* *Nitrosotalea devanaterra*” having high affinities for ${NH}_{4}^{+}/{NH}_{3}$ that rival or exceed some heterotrophs (*K*_m_ = 0.13 μM total ${NH}_{3}/{NH}_{4}^{+}$; Martens-Habbena et al., 2009; Lehtovirta-Morley et al., 2011). These findings have provided the impetus to identify the soil factors that affect the relative contributions of AOA and AOB to soil nitrification. With this information in hand, it might be possible to determine if there is a relationship between the relative contributions of AOA and AOB to nitrification and the amounts of ${NH}_{4}^{+}$ nitrified and immobilized, respectively.

## IDENTIFICATION OF “POTENTIALLY GROWTH-ACTIVE” SUBPOPULATIONS BY TARGETING NUCLEIC ACID SYNTHESIS

We have raised the issue of why it might be important to delineate between substrate-responsive and growth-active subpopulations and hypothesized that the latter are most likely candidates for N immobilizing activity [see How Does Gross Mineralization of N (N_min_) and Gross Immobilization of N (N_assim_) Occur in the Same Soil Volume?, Substrate-Responsive Population: Growth Ready or Not?, and What Controls the Outcome of Competition for ${NH}_{3}/{NH}_{4}^{+}$ Between Heterotrophs and NH_3_ Oxidizers, and Among Different NH_3_ Oxidizers]. We have selected three papers from recent literature where nucleic acid analytical approaches were used to identify potentially growth-active subpopulations in soil communities.

### BrdU-LABELED DNA

McMahon et al. (2011) used the thymidine analog, 5-bromo-3-deoxyuridine (BrdU), to identify the growth responsive subpopulation in Arctic tundra shrub and tussock associated soils at different times of the year. BrdU was added to soil along with a small quantity of various monomeric substrates (100 μg C/g soil of either glucose, glutamate, or vanillin) sufficient to “act as a tracer of extant microbial activity” and yet insufficient to stimulate overall CO_2_ production. Winter and summer soils were incubated at -2°C for 28 days, and at 4°C for 2 days, respectively. After incubation DNA was extracted, BrdU-labeled DNA recovered by immunocapture, and 16S rRNA genes PCR amplified and analyzed with the T-RFLP approach. None of the BrdU-labeled terminal restriction fragments (T-RFs) recovered from winter samples of shrub soil matched up with the T-RFs representing the total winter community suggesting that “winter growth-active” organisms were minor components of the community. Although there were more T-RF overlaps between the active and total communities of the summer shrub soil samples, many “active” T-RFs in the summer samples did not overlap with the total summer community. Because the balance between N cycling activities of tundra soils shifts between winter (net N mineralization dominant) and summer (net N immobilization dominant), this study serves to illustrate the importance of determining the amounts of growth that occur in the growth-active subpopulations during the winter and summer periods. This will impact the amount of N immobilized and the overall balance of the N cycle.

### ^18^O-LABELED DNA

Aanderud and Lennon (2011) targeted the growth-active members of a soil population carrying out DNA synthesis after rewetting a soil with ^18^O-labeled H_2_O. Labeled H_2_O was added to field dry soil to raise water content from 0.05 to 0.25 g H_2_O/g. Samples were incubated for 72 h, DNA was extracted, and “heavy” DNA separated from “light” DNA by gradient density centrifugation. Pyrosequencing was used to compare the composition of 16S rRNA gene sequences recovered from the light DNA at time zero with that of heavy DNA extracted after 72 h of incubation. The contributions of some phyla to the heavy DNA fraction increased by about 10% (Alpha-, Beta-, and Gamma-proteobacteria), whereas the contribution of other taxa declined (Chloroflexi, Delta-proteobacteria) implying that selective growth had occurred. Because Alpha-proteobacteria are generally more abundant than Gamma-proteobacteria in bulk soils (Lauber et al., 2009; Rousk et al., 2010a) the similar relative population increases during the rewet, implies that the amount of biomass increase and N immobilized by Alpha-proteobacteria might be greater than by Gamma-proteobacteria.

### RELATIVE QUANTITIES OF rRNA VERSUS rRNA GENE SEQUENCES

Baldrian et al. (2011) extracted DNA and RNA from both the litter and humus layers of a spruce forest soil and used a pyrosequencing approach to compare the relative amounts of 16S rRNA and 16S rRNA gene sequences of bacteria, and compared the relative amounts of RNA and DNA sequences of the ITS1–5.8S–ITS2 region of fungi. Whereas several abundant fungal OTUs identified from DNA sequences were also highly enriched in RNA sequences, a substantial percentage of fungal OTUs (18%) were only found in the RNA community, implying that less abundant fungi might be potentially growth-active. This idea was further supported by the most abundant “cellobiohydrolase” (cbh) transcripts originating from less abundant fungi.

## IDENTIFYING THE RELATIVE CONTRIBUTIONS OF DIFFERENT MICROBIAL GROUPS TO N CYCLE ACTIVITIES USING SELECTIVE PROTEIN SYNTHESIS INHIBITORS

Several studies have attempted to tease apart the relative contributions of different groups of soil microorganisms to N cycling activities by targeting protein synthesis (Castaldi and Smith, 1998; Laughlin and Stevens, 2002; Tungaraza et al., 2003; Castaldi, 2005; Myrold and Posavatz, 2007; Boyle et al., 2008; Rousk et al., 2008, 2009, 2010b). In this section, we highlight two published papers where protein synthesis inhibitory antibiotics were used to differentiate (a) the contributions of bacteria and fungi to ${NH}_{4}^{+}$ uptake and N mineralization, and (b) the relative contributions of AOA and AOB to soil NH_3_ oxidizing potential.

### FUNGAL AND BACTERIAL CONTRIBUTIONS TO N CYCLING BASED UPON USE OF ANTIBIOTICS AND ^15^N ISOTOPE POOL DILUTION MEASUREMENTS

Boyle et al. (2008) compared the effects of the bacterial and fungal protein synthesis inhibitors, bronopol and cycloheximide on gross rates of N cycling in forest soils using the ^15^N isotope pool dilution method. Bronopol completely stopped ${NH}_{4}^{+}$ consumption in high N soils implying that bacteria dominated ${NH}_{4}^{+}$ uptake, and that fungi were not involved in ${NH}_{4}^{+}$ consumption even when bacterial growth was inhibited. In contrast, bronopol had no effect on ${NH}_{4}^{+}$ consumption in a low N soil under the conifer Douglas-fir implying that bacteria were not involved in ${NH}_{4}^{+}$ consumption, or, if they were, bacterial consumption could be interchangeably replaced by fungal consumption. Soil ammonification was increased by bronopol at the high N site, implying that when bacterial-dependent ${NH}_{4}^{+}$ consumption was stopped, organic N mineralization continued and ${NH}_{4}^{+}$ accumulated. Cycloheximide consistently increased both ammonification and ${NH}_{4}^{+}$ consumption, implying that when fungal protein synthesis was stopped more ${NH}_{4}^{+}$ was made available for bacterial consumption. These data point to fungi likely consuming organic N for protein synthesis. Clearly much remains to be learned about the factors influencing the relative contributions of bacteria and fungi to processing organic and inorganic N sources, and what factors influence the extent the two types of microorganisms operate independently or compete for N resources.

### THE RELATIVE CONTRIBUTIONS OF AMMONIA-OXIDIZING ARCHAEA AND BACTERIA TO SOIL NITRIFICATION POTENTIALS

Although the ability of AOA to oxidize NH_3_ and grow autotrophically in soil has been well established (Offre et al., 2009; Zhang et al., 2010; Verhamme et al., 2011), and that AOA dominate ${NH}_{3}/{NH}_{4}^{+}$ oxidation under strongly acidic soil conditions (Stopnisek et al., 2010; Lehtovirta-Morley et al., 2011), little is known about what influences the relative contributions of AOA and AOB to soil nitrification under most other soil conditions. Taylor et al. (2010) developed a short-term assay based upon selective inactivation of ammonia monooxygenase (AMO) by acetylene. After removal of acetylene the recovery of the nitrification potential (RNP) was followed in the presence and absence of protein synthesis inhibitors/antibiotics and/or fungicides. The success of the assay is based upon determining the fraction of the RNP that occurs within 24–36 h post acetylene removal in the presence of protein synthesis inhibitors and which is assumed to be due to AOA (Taylor et al., 2010). This study showed that in recently N fertilized cropped soils with high NP, the majority of RNP activity is due to AOB, and that in pasture and grassland soils with lower NP activity, RNP is due primarily to AOA or to a mixture of AOA and AOB. A subsequent study has shown that the factors controlling the relative contributions are complex with cropping treatment, soil conditions, and ${NH}_{4}^{+}$ availability influencing their relative contributions in the field (Taylor et al., 2012). Further studies are required that combine measurements of the relative contributions of AOA and AOB to nitrification with those of N immobilization to determine if AOA/AOB contributions affect the N/I ratio.

## CONCLUDING REMARKS

Over the past 25 years considerable effort has been spent refining our understanding of how the physical and chemical properties of the soil environment interact with microbial communities to maintain an overall balance between the mineralization and immobilization of soil N. During the past 10 years considerable information has been generated on the overall diversity and composition of soil microbial communities. In this review, we have selected a few recent publications that are focused upon the activities of subpopulations of soil microbes and discussed how the implications of this work may lead to a better understanding of N cycling. Clearly, this mini-review is not meant to be all inclusive, but we hope that the readers’ attention has been drawn to “soil phenomena” into which they might “dig” and “unearth” an increased level of understanding about the physiology and growth response behaviors of soil subpopulations and how they influence the balance between mineralization, nitrification, and immobilization of soil N.

## Conflict of Interest Statement

The authors declare that the findings cited from their own research in this minireview was conducted in the absence of any commercial or financial relationships that could be construed as a potential conflict of interest.
